# When Emotions Matter: Focusing on Emotion Improves Working Memory Updating in Older Adults

**DOI:** 10.3389/fpsyg.2017.01565

**Published:** 2017-09-15

**Authors:** Natalie Berger, Anne Richards, Eddy J. Davelaar

**Affiliations:** Department of Psychological Sciences, Birkbeck, University of London London, United Kingdom

**Keywords:** working memory, updating, aging, task relevance of emotion, *n*-back task

## Abstract

Research indicates that emotion can affect the ability to monitor and replace content in working memory, an executive function that is usually referred to as updating. However, it is less clear if the effects of emotion on updating vary with its relevance for the task and with age. Here, 25 younger (20–34 years of age) and 25 older adults (63–80 years of age) performed a 1-back and a 2-back task, in which they responded to younger, middle-aged, and older faces showing neutral, happy or angry expressions. The relevance of emotion for the task was manipulated through instructions to make match/non-match judgments based on the emotion (i.e., emotion was task-relevant) or the age (i.e., emotion was task-irrelevant) of the face. It was found that only older adults updated emotional faces more readily compared to neutral faces as evidenced by faster RTs on non-match trials. This emotion benefit was observed under low-load conditions (1-back task) but not under high-load conditions (2-back task) and only if emotion was task-relevant. In contrast, task-irrelevant emotion did not impair updating performance in either age group. These findings suggest that older adults can benefit from task-relevant emotional information to a greater extent than younger adults when sufficient cognitive resources are available. They also highlight that emotional processing can buffer age-related decline in WM tasks that require not only maintenance but also manipulation of material.

## Introduction

The ability to maintain only relevant information while replacing outdated content in working memory (WM) is crucial for adaptive functioning and gives evolutionary advantage as new information, that may be potentially harmful, will be dealt with quickly. This ability is usually referred to as updating and is thought to represent one core executive function among three (Miyake et al., 2000; Miyake and Friedman, 2012) that are believed to form the central executive in Baddeley and Hitch's (1974) WM system. In everyday life, information is often emotional and researchers have started investigating emotional updating, with some even linking it to efficient emotion regulation (Levens and Gotlib, 2010; Pe et al., 2013a,b). However, despite evidence that emotion can have both enhancing and impairing effects on executive functions depending on its relevance for the on-going task (Pessoa, 2009, 2015, 2017), task relevance of emotion was often not considered in previous studies on emotional updating. Moreover, it is not clear whether task-relevant and task-irrelevant emotion affects updating similarly in younger and older adults as aging is not only associated with changes in WM updating (Van der Linden et al., 1994; Hartman et al., 2001; Salthouse et al., 2003; De Beni and Palladino, 2004; Chen and Li, 2007; Schmiedek et al., 2009) but also in preference for emotional material (for reviews, see Scheibe and Carstensen, 2010; Reed and Carstensen, 2012). This research aims to close the empirical gap by assessing younger and older adults' WM updating in the presence of task-relevant and task-irrelevant emotion.

### Task relevance modulates effects of emotion on executive functions

Emotional information was found to have mixed effects on WM performance, with some studies reporting facilitating effects (Erk et al., 2007; Levens and Phelps, 2008; Lindström and Bohlin, 2011; Exp. 1, Pessoa et al., 2012) and others showing impairing effects (Kensinger and Corkin, 2003; Gotoh, 2008; Lindström and Bohlin, 2012; Exp. 2, Pessoa et al., 2012; Kopf et al., 2013), particularly when task-irrelevant emotion was used (Dolcos and McCarthy, 2006; Hart et al., 2012; Dolcos et al., 2013; Iordan et al., 2013). Task relevance of emotion has indeed been found to play an important role in the effects of emotion on executive functions such as updating. According to the dual competition model (Pessoa, 2009, 2015, 2017), task-relevant emotion improves executive functions through recruitment of additional processing resources, whereas task-irrelevant emotion impairs them through detraction of resources from the task.

In previous studies on emotional updating, however, task relevance of emotion was often not considered. Many have used the *n*-back task (Kirchner, 1958), in which participants have to monitor a sequence of items that are presented one at a time. For each item, one of two keys is pressed to indicate whether it is the same as (match) or different from (non-match) the one presented *n* positions back in the sequence. In many *n*-back studies with emotional material, participants were asked to make match/non-match responses without being told whether to base their response on the item's emotion or not (e.g., Kensinger and Corkin, 2003; Döhnel et al., 2008; Lindström and Bohlin, 2011; Phillips et al., 2011; Kopf et al., 2013; Richter et al., 2013; Schoofs et al., 2013; Weigand et al., 2013). With such unspecific instructions, it is not clear whether all participants responded to the same stimulus features. For instance, participants could respond to the identity rather than the emotional expression of facial stimuli, which would make emotion a task-irrelevant feature. These studies reported mixed findings with some showing improved performance for emotional relative to neutral items (Döhnel et al., 2008; Lindström and Bohlin, 2011; Phillips et al., 2011), whereas others showed impaired performance for negative relative to neutral or positive items (Kensinger and Corkin, 2003; Kopf et al., 2013). It is possible that this mixed pattern of results was due to the fact that effects of task-relevant and task-irrelevant emotion were intermixed.

A more coherent pattern of results emerged in *n*-back studies with explicit instructions regarding the items' emotional features. Studies with task-relevant emotion typically showed an improving effect of positive material and particularly happy faces on WM updating (Levens and Gotlib, 2010, 2012; Pe et al., 2013a; Cromheeke and Mueller, 2015). Other studies used explicitly irrelevant neutral or emotional distractors with non-emotional targets such as letters or digits (e.g., Ladouceur et al., 2009; Bakvis et al., 2010; Marx et al., 2011; Bertocci et al., 2012; Mullin et al., 2012; Miendlarzewska et al., 2013; Ozawa et al., 2014). Usually, no differences between the effects of emotional (negative or positive) and neutral distractors were found (Mullin et al., 2012; Miendlarzewska et al., 2013; Ozawa et al., 2014), unless the emotional distractors were highly arousing (Marx et al., 2011). Overall, it appears that task-relevant and particularly positive emotion can enhance updating, whereas task-irrelevant emotional content does not impair performance if it is low in arousal. However, these studies focused on younger adults, whereas emotional updating in older adults has received little attention to this date, despite evidence that emotion-cognition interactions change in aging.

### Age-related changes in WM updating and emotional functioning

Aging is associated with impairments in WM updating (Van der Linden et al., 1994; Hartman et al., 2001; Salthouse et al., 2003; De Beni and Palladino, 2004; Chen and Li, 2007; Schmiedek et al., 2009). For instance, *n*-back studies have shown that older adults were more susceptible to interference from task-irrelevant lures than younger adults (De Beni and Palladino, 2004; Schmiedek et al., 2009), suggesting they had greater difficulty in updating the relevant *n*-back sequence and relied more on familiarity. Aging is also associated with changes in emotional functioning. According to the socioemotional selectivity theory (SST; Carstensen, 1993), older adults allocate more cognitive resources to emotional and more specifically to positive material than younger adults to enhance their well-being, resulting in an age-related “positivity effect” (for reviews, see Scheibe and Carstensen, 2010; Reed and Carstensen, 2012). It was found that the emotional bias in aging can be eliminated under conditions of high cognitive load (Mather and Knight, 2005), suggesting that age-related changes in emotion-cognition interactions are due to controlled, resource-demanding processes. These age-related changes in WM updating and emotional functioning are likely to influence emotional updating in aging.

Moreover, there is evidence that task-relevant emotion can improve WM performance to a greater extent in older than in younger adults. In a delayed-response task, age-related impairments were found when the brightness of two neutral pictures was compared, but not when the emotional intensity of two emotional pictures was compared (Mikels et al., 2005). Similarly, age-related impairments were found when neutral but not when emotional words were used in a modified version of the operation WM span test, which requires participants to maintain words while solving mathematical operations (Mammarella et al., 2013). These findings suggest that age-related differences in WM can be reduced or eliminated when emotional stimuli are used. However, as these studies focused on *maintenance* of material, it is less clear whether emotion can boost older adults' performance in more complex WM tasks that require additional *manipulation* of information, such as WM updating tasks. In contrast, there is also evidence for particularly impairing effects of task-irrelevant emotion on WM in aging (Wurm et al., 2004; Borg et al., 2011; Truong and Yang, 2014). For instance, emotional and neutral words were used as targets or distractors in a delayed-response WM task (Truong and Yang, 2014). Younger and older adults had to indicate whether subsequently presented probes had been targets. Both age groups were faster and more accurate when emotional words were relevant targets but only older adults were less accurate when emotional words were irrelevant distractors. Interference from task-irrelevant emotional material was also found for older but not younger adults in two emotional Stroop tasks (Wurm et al., 2004). The authors suggested that fewer cognitive resources made older adults more susceptible to disruptive effects of automatic activation in the presence of emotional distractors.

Overall, these findings suggest that the facilitating and impairing effects of emotion can be more pronounced in aging and that the modulatory effects of emotion appear to depend on its relevance for the task. Crucially, facilitating and impairing effects might be intermixed in updating studies with nonspecific instructions regarding the emotional item features. For instance, Döhnel et al. (2008) used emotional pictures in an *n*-back task and did not find age-related differences in emotional updating in older relative to younger adults. However, unspecific match/non-match instructions were used and participants may have focused on different item features or used different strategies for different valences. Such inconsistencies might have clouded the effects of emotion and age on WM updating.

### The present study

So far, only one study has systematically varied the task relevance of emotion in an *n*-back study on aging. Pehlivanoglu et al. (2014) asked younger and older adults to update emotional and neutral faces in an *n*-back task and to base their decision on either the facial expression, the identity, or on both. Although this design allowed testing (un)binding processes in both age groups, the effects of task-relevant (i.e., expression condition) or task-irrelevant emotion (i.e., identity condition) on updating in the two age groups were not tested. The aim of the present research was therefore to directly compare older and younger adults' *n*-back performance in the presence of task-relevant or irrelevant emotion. Three further aspects were considered: Firstly, three levels of emotion (happy, neutral, angry) were used in all comparisons to evaluate the effect of valence vs. arousal on updating. Secondly, two levels of load were included to assess whether emotional updating is affected differently by load in aging. Thirdly, the factor trial type (match, non-match) was included in the analysis as evidence suggests that emotion can interact with trial type (Kensinger and Corkin, 2003; Levens and Gotlib, 2010, 2012). For instance, Levens and Gotlib (2010, 2012) reported that replacing but not matching happy relative to neutral faces was slower in healthy adults. Moreover, only non-match trials require the actual updating (i.e., replacement or overwriting) of old representations with new ones (Verhaeghen and Basak, 2005; Chen et al., 2008). Including the factor trial type therefore allowed to assess whether emotion affects the updating process on non-match trials or shared processes across trial types (e.g., emotion processing).

The following hypotheses were tested: (1) Task-relevant and particularly positive emotion will improve WM updating in terms of higher accuracy and faster RTs, with no such effect for task-irrelevant emotion. (2) This facilitating effect will be more pronounced in older than in younger adults but only under low-load (i.e., 1-back) and not under high-load conditions (i.e., 2-back), as older adults show a bias for emotional and particularly positive material (Carstensen and Mikels, 2005; Scheibe and Carstensen, 2010; Reed and Carstensen, 2012), which is eliminated by load (Mather and Knight, 2005). (3) Task-irrelevant and particularly negative emotion will impair updating performance in older but not in younger adults as shown in previous WM studies (Wurm et al., 2004; Borg et al., 2011; Truong and Yang, 2014). Should the predicted interactions including the factors emotion and age be observed for non-match but not for match trials, this would indicate that emotion and age affect the actual updating process needed on non-match trials. Should the effects be the same for both trial types, this would indicate that emotion and age affect general processes needed in an *n*-back task.

## Materials and methods

### Participants

Twenty-five younger (20–34 years old) and 25 older adults (63–80 years old) participated in the study (see Table 1 for participant characteristics). The sample size was determined on the basis of past related work with similar experimental conditions (Pehlivanoglu et al., 2014). The younger adults were students at Birkbeck College and received course credits or a small fee for participating. The older adults were recruited from the University of the Third Age in the Greater London area and received a small fee for taking part.

**Table 1:** Participant characteristics.

	**Younger adults**		**Older adults**		**Group difference**	
	***M***	***SD***	***M***	***SD***	***t***	***p***
Age (years)	25.32	4.14	68.80	5.94	−30.04	<0.001
Gender (female/male)	17/8		18/7			
Education (years)	17.30	2.52	15.70	2.33	2.33	0.024
NART verbal IQ	110.54	5.04	120.06	4.52	−5.38	<0.001
Digit symbol test	64.64	12.09	58.06	11.73	1.78	0.082
BDI II	5.84	5.76	3.84	2.64	1.58	0.121
STAI trait anxiety	32.16	9.02	29.96	9.58	0.84	0.407
MMSE			29.20	0.91		

*NART, The National Adult Reading Test; BDI II, Beck Depression Inventory II; STAI, State-Trait Anxiety Inventory; MMSE, Mini-Mental State Examination*.

All participants were community-dwelling and reported to be in good health and to have normal or corrected-to-normal vision and hearing. They were also pre-screened for history of psychiatric or neurological disorders. Older participants had a score of 27 or above on the Mini-Mental State Examination (MMSE; Folstein et al., 1975), a screening for cognitive impairments. As can be seen in Table 1, older adults reported fewer years of schooling than younger adults. Consistent with typical profiles in the literature, older adults had better verbal knowledge than in younger adults as assessed with the National Adult Reading Test (NART; Nelson and Willison, 1991). They also scored (marginally) lower on the Digit Symbol Substitution Test from the WAIS-R (Wechsler, 1955) than younger adults, indicating slower processing speed. This study was carried out in accordance with the recommendations of the ethics board of Birkbeck, University of London, with written informed consent from each participant. All participants gave written informed consent in accordance with the Declaration of Helsinki.

### Materials

Stimuli consisted of 72 images of faces from the FACES database (Ebner et al., 2010), a validated set of color photographs of naturalistic, front-facing faces of different ages. In a pilot study, 10 younger (21–32 years old; *M* = 27.80, *SD* = 3.12) and 10 older adults (66–76 years old; *M* = 71.27, *SD* = 3.13) rated 234 preselected faces on valence and arousal and estimated the age of each face. From this set, 72 faces (24 happy, 24 angry, 24 neutral expressions) with the highest agreement between younger and older raters were selected for the main experiment. Age group and sex of the face models were balanced in each emotion category, resulting in eight pictures per age group and emotion category with four male and four female faces. Each picture showed a unique individual. For counterbalancing purposes, two face sets were created that had similar arousal and valence levels [all *ts*_(19)_ < 1.30, *ps* < 0.208; see Supplemental Materials for more details].

### Procedure

After giving informed consent and providing demographics, a short computer-based visual acuity test (Bach, 1996) was administered at a distance of 65 cm to ensure that vision was in the normal range. Participants were then asked to remain at this distance to the screen and to complete the computerized *n*-back tasks, starting with a 0-back task followed by the 1-back and 2-back tasks in consecutive order. Short breaks were offered between tasks. After the *n*-back tasks, participants completed the Digit Symbol Substitution Test (Wechsler, 1955), the NART (Nelson and Willison, 1991), the Beck Depression Inventory (BDI-II; Beck et al., 1988) and the A-Trait version of the State-Trait Anxiety Inventory (STAI; Spielberger et al., 1983). Older adults additionally completed the MMSE (Folstein et al., 1975). Participants were debriefed at the end of the session, which lasted 1.5 to 2 h.

### *N*-back tasks

The *n*-back tasks were prepared and presented using E-Prime Version 2.0.10.353 (Schneider et al., 2002) on a 24-inch computer screen with a resolution of 1,920 × 1,200 pixels. The experiment comprised two tasks: (i) a 1-back task, in which the current face was compared with the face presented one trial earlier and (ii) the 2-back task, in which the current face was compared with the face presented two trials earlier. They were completed in consecutive order and preceded by a 0-back task, which was included to familiarize participants with the procedure and the stimuli and in which the current face was compared to a target label. In each task, participants were instructed to respond to match trials by pressing “same” and to non-match trials by pressing “different” based on the emotional expression (angry vs. neutral vs. happy; emotion task-relevant) or the age of the face (young vs. middle-aged vs. old; emotion task-irrelevant). In the instructions to respond to the age, emotion was not mentioned (i.e., they were not told to ignore emotion). If participants are instructed to ignore distractors, then this can become part of the goal structure and may result in paradoxical effects as shown in a flanker task (Davelaar, 2012). People could still incorporate an ignore-emotion goal due to the within-subject manipulation of the relevance of emotion, but it was expected to be minimal.

**Figure 1:**
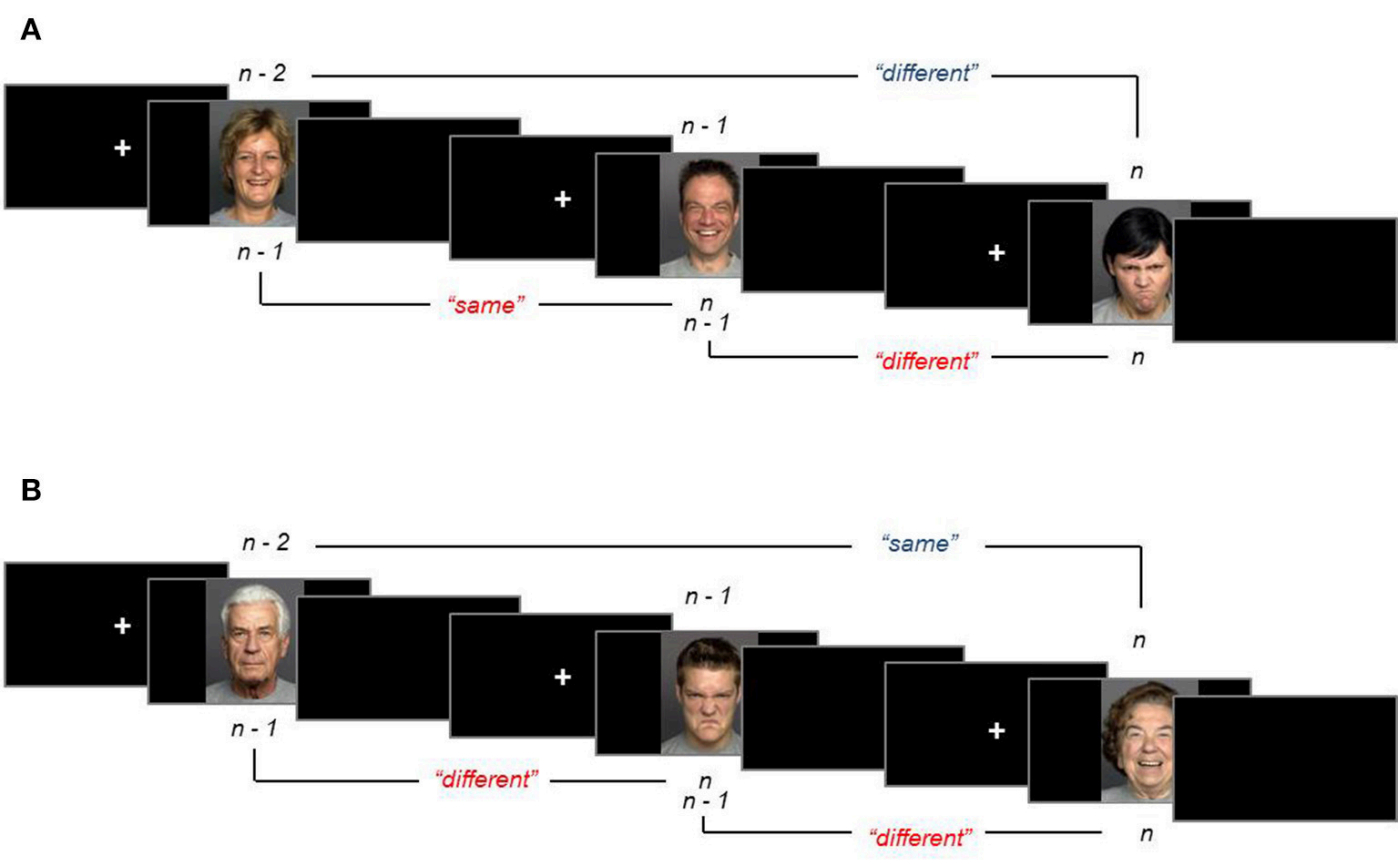
Examples of the expression task with task-relevant emotion **(A)** and the age task with task-irrelevant emotion **(B)**, including correct responses. Correct responses in the 1-back task are shown in red font at the bottom of each panel and those in the 2-back task are shown in blue font at the top of each panel.

In each task, a fixation cross appeared for 500 ms and was replaced by a face for 2,000 ms after which a blank screen was presented for 200 ms. Participants were instructed to respond by pressing one of two labeled buttons (“S” for same, “D” for different). On a regular PC keyboard, the buttons “1” and “2” of the numeric keypad were used and participants were instructed to leave the index finger and middle finger on the two buttons for the duration of the task. The face remained on the screen for the full 2,000 ms even after response. All 72 stimuli were included in the preparatory 0-back task; they were separated into six blocks of 12 items and presented once. Before each block, an emotion label (“angry,” “neutral,” or “happy”), or an age label (“young,” “middle-aged,” or “old”) was presented. Participants compared the emotional expression or age of the face with the label, and the order of expression and age blocks was randomized. Half of the stimuli were then used in the 1-back and the other half in the 2-back task. Assignment of the two stimulus sets to the 1-back or 2-back task was counterbalanced. In each *n*-back task, emotion was task-relevant for half of the blocks and task-irrelevant for the other half. In each block, 50% of trials were match trials and 50% were non-match trials. Participants were instructed to respond as accurately and quickly as possible. See Figure 1 for example trials of the 1-back and 2-back tasks.

#### 1-back task

The 1-back task consisted of 220 trials that were divided into four blocks of 55 items. The task was preceded by 20 practice trials. Expression and age blocks were presented in alternating order and the start with either an expression or age block was counterbalanced across participants. For each block, participants were instructed to view the first face without pressing a key; from the second face onwards, participants were instructed to respond, resulting in 54 usable trials per block. Each item was presented on average 6 times.

#### 2-back task

The 2-back task consisted of 304 trials that were separated into eight blocks of 38 items. The task was preceded by 24 practice trials. Counterbalancing was the same as in the 1-back task and the same decisions were made, but this time, participants were required to compare the current face with the face presented two trials earlier. For the first two faces in each block, participants viewed the faces without pressing a key; from the third face on, participants were instructed to respond, resulting in 36 usable trials per block. Each item was presented on average 8 times.

### Statistical analysis

Responses and RTs were recorded for each trial and the percentages of hits (correct match) and false positives (incorrect match) were calculated for each condition. RTs were analyzed as the primary dependent variable (Kensinger and Corkin, 2003; Levens and Gotlib, 2010, 2012). RTs faster than 200 ms or 2.5 standard deviations above or below the group mean for the 1-back or 2-back task were excluded, resulting in an exclusion of an average of 2.4% and 1.62% of trials per task for younger and older adults, respectively. Median RTs for correct trials were then calculated for each condition. To obtain corrected measures of recognition, hits and false positives were used to calculate *A'*, a measure of detection sensitivity (Grier, 1971), for each condition. A non-parametric index was chosen as it is robust even with relatively low numbers of observations per unique condition (Grier, 1971) as in the present design.

Statistical analyses were conducted with SPSS 22 (IBM Corp., Armonk, NY). Detection sensitivity scores were submitted to a mixed factors ANOVA including the within-subjects factors load (1-back vs. 2-back), task (expression vs. age), and emotion (angry vs. neutral vs. happy) as well as the between-subjects factor of age group (younger vs. older). RTs for correct responses were analyzed with the same factors as above plus the additional within-subjects factor of trial type (match vs. non-match). Bonferroni-corrected post-hoc *t*-tests were performed to follow up significant effects. All tests were two-tailed with α = 0.05. All statistical results for detection sensitivity and RTs are presented in Table 2, and separate results for the expression and age tasks are reported in Table 3. Results that were relevant for the hypotheses are reported in the text below.

**Table 2:** Statistical results for *n*-back performance.

**Measure**	***F***	***MSE***	***p***	**Partial η^2^**
**DETECTION SENSITIVITY**				
Load	60.47	0.02	<0.001^***^	0.56
Task	35.22	0.01	<0.001^***^	0.42
Emotion	4.30	0.01	0.016^*^	0.08
Age group	9.08	0.05	0.004^**^	0.16
Load × Task	3.99	0.01	0.051	0.08
Load × Emotion	0.65	<0.01	0.526	0.01
Load × Age group	7.75	0.02	0.008^**^	0.14
Task × Emotion	19.15	0.01	<0.001^***^	0.29
Task × Age group	1.14	0.01	0.291	0.02
Emotion × Age group	0.56	0.01	0.571	0.01
Load × Task × Emotion	1.04	<0.01	0.358	0.02
Load × Task × Age group	7.39	0.01	0.009^**^	0.13
Load × Emotion × Age group	0.39	<0.01	0.681	0.01
Task × Emotion × Age group	0.23	0.01	0.791	0.01
Load × Task × Emotion × Age group	0.06	<0.01	0.944	<0.01
**CORRECT RESPONSE TIME**				
Load	31.10	76,418	<0.001^***^	0.39
Task	14.21	15,855	<0.001^***^	0.23
Trial type	54.97	17,059	<0.001^***^	0.53
Emotion	31.43	5,366	<0.001^***^	0.40
Age group	69.39	14,712	<0.001^***^	0.59
Load × Task	8.52	7,988	0.005^**^	0.15
Load × Trial type	11.22	11,391	0.002^**^	0.19
Load × Emotion	4.45	7,494	0.014^*^	0.09
Load × Age group	11.16	76,418	0.002^**^	0.19
Task × Trial type	2.07	8,417	0.157	0.04
Task × Emotion	23.32	7,439	<0.001^***^	0.33
Task × Age group	3.12	15,855	0.084	0.06
Trial type × Emotion	31.83	6,086	<0.001^***^	0.40
Trial type × Age group	0.42	17,059	0.522	0.01
Emotion × Age group	0.18	5,366	0.535	<0.01
Load × Task × Trial type	4.44	5,681	0.040^*^	0.09
Load × Task × Emotion	0.32	5,004	0.728	0.01
Load × Task × Age group	3.70	7,988	0.060	0.07
Load × Trial type × Emotion	1.44	4,268	0.243	0.03
Load × Trial type × Age group	0.01	11,391	0.909	<0.01
Load × Emotion × Age group	0.34	7,494	0.715	0.01
Task × Trial type × Emotion	36.15	7,024	<0.001^***^	0.43
Task × Trial type × Age group	0.39	8,417	0.534	0.01
Task × Emotion × Age group	0.85	7,439	0.432	0.02
Trial type × Emotion × Age group	2.85	6,068	0.063	0.06
Load × Task × Trial type × Emotion	1.24	4,689	0.294	0.03
Load × Task × Trial type × Age group	0.61	12,178	0.520	0.01
Load × Task × Emotion × Age group	1.36	5,004	0.261	0.03
Load × Trial type × Emotion × Age group	1.50	4,268	0.228	0.03
Task × Trial type × Emotion × Age group	1.97	7,024	0.145	0.04
Load × Task × Trial type × Emotion × Age group	3.46	4,689	0.035^*^	0.07

*** *p < 0.001*,

** *p < 0.01*,

* *p < 0.05*.

**Table 3:** Statistical results for *n*-back performance in expression and age tasks.

	**Expression task (i.e., emotion relevant)**				**Age task (i.e., emotion irrelevant)**			
**Measure**	***F***	***MSE***	***p***	**Partial η^2^**	***F***	***MSE***	***p***	**Partial η^2^**
**DETECTION SENSITIVITY**								
Load	57.27	0.01	<0.001^***^	0.54	32.79	0.01	<0.001^***^	0.41
Emotion	21.17	0.01	<0.001^***^	0.31	3.51	0.01	0.034^*^	0.07
Age group	5.12	0.03	0.028^*^	0.10	10.67	0.03	0.002^*^	0.18
Load × Emotion	1.00	<0.01	0.371	0.02	0.75	0.01	0.474	0.02
Load × Age group	13.88	0.01	0.001^**^	0.22	1.00	0.01	0.323	0.02
Emotion × Age group	0.34	0.01	0.714	0.01	0.44	0.01	0.644	0.01
Load × Emotion × Age group	0.19	<0.01	0.830	<0.01	0.23	0.01	0.793	0.01
**CORRECT RESPONSE TIME**								
Load	47.39	34,276	<0.001^***^	0.50	16.36	50,130	<0.001^***^	0.25
Trial type	60.83	9,951	<0.001^***^	0.56	22.53	15,525	<0.001^***^	0.32
Emotion	45.41	7,412	<0.001^***^	0.49	1.02	5,393	0.363	0.02
Age group	59.06	189,205	<0.001^***^	0.56	74.42	179,730	<0.001^***^	0.61
Load × Trial type	2.00	9,874	0.164	0.04	18.51	7,197	<0.001^***^	0.28
Load × Emotion	2.61	6,833	0.079	0.05	3.02	5,665	0.053	0.06
Load × Age group	17.51	34,276	<0.001^***^	0.27	5.64	50,130	0.022^*^	0.11
Trial type × Emotion	51.40	8,667	<0.001^***^	0.52	0.48	4,443	0.620	0.01
Trial type × Age group	1.01	9,951	0.320	0.02	0.02	15,525	0.880	<0.01
Emotion × Age group	0.82	7,412	0.442	0.02	0.22	5,393	0.806	<0.01
Load × Trial type × Emotion	1.01	9,157	0.393	0.02	0.11	5,233	0.464	0.02
Load × Trial type × Age group	0.67	9,874	0.416	0.01	0.57	7,197	0.453	0.01
Load × Emotion × Age group	1.17	6,833	0.315	0.02	0.24	5,665	0.788	0.01
Trial type × Emotion × Age group	3.06	8,667	0.049^*^	0.06	0.95	4,443	0.390	0.02
Load × Trial type × Emotion × Age group	4.99	3,724	0.009^**^	0.09	0.77	5,233	0.464	0.02

*** *p < 0.001*,

** *p < 0.01*,

* *p < 0.05*.

## Results

### Detection sensitivity

**Figure 2:**
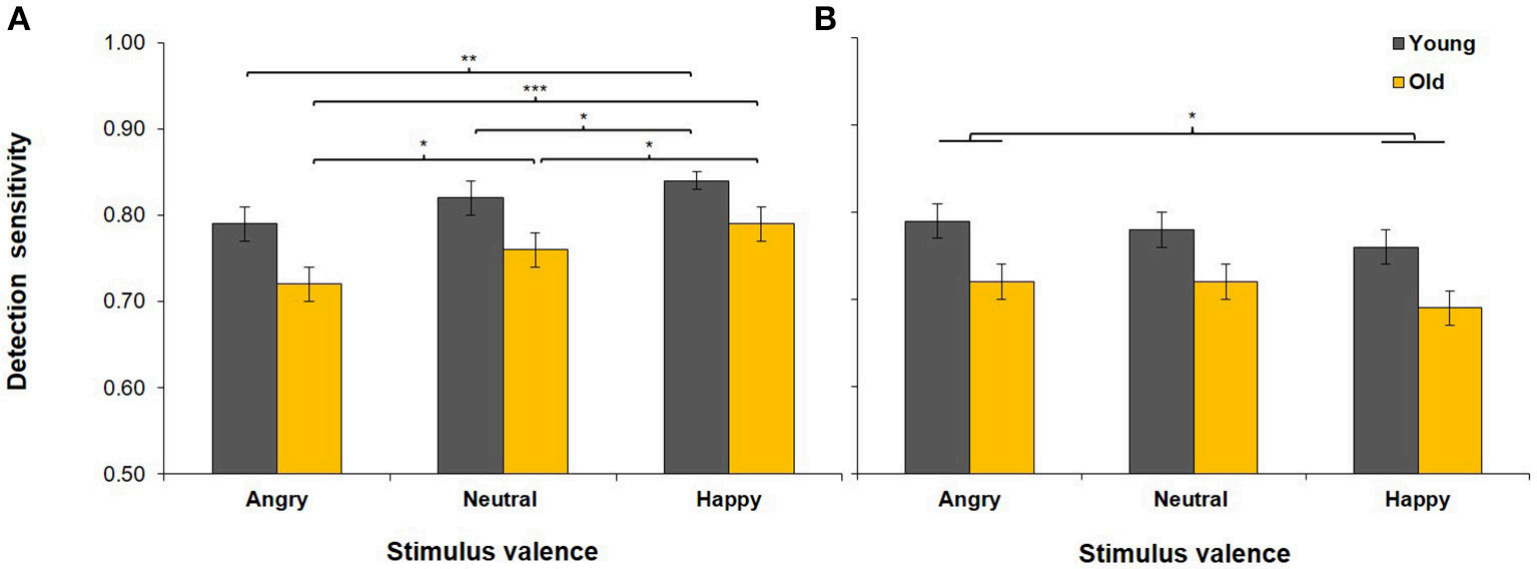
Detection sensitivity in younger and older adults in the expression task (i.e., participants responded to the emotional expression of the faces; **A**) and in the age task (i.e., participants responded to the age of the faces; **B**), collapsed across the 1-back and the 2-back loads. Error bars represent SEM. ^***^p < 0.001, ^**^p < 0.01, ^*^p < 0.05.

Detection sensitivity scores in both age groups in the expression and age tasks are presented in Figure 2. The manipulation of load was successful as indicated by a main effect of load (see Table 2), with lower overall detection sensitivity in the 2-back (*M* = 0.82, *SD* = 0.06) than in the 1-back task (*M* = 0.73, *SD* = 0.10). The four-way omnibus ANOVA yielded a significant task × emotion interaction, which qualified the main effect of emotion. Separate analyses for detection sensitivity in the expression and the age tasks were conducted to follow up this interaction (see Table 3).

#### Effects of task-relevant emotion

As predicted by hypothesis 1, there was a main effect of task-relevant emotion in the expression task (see Table 3), with greater detection sensitivity for happy (*M* = 0.82, *SD* = 0.09) than for neutral (*M* = 0.79, *SD* = 0.10), *t*_(49)_ = 2.95, *p* = 0.005, or angry faces (*M* = 0.75, *SD* = 0.10), *t*_(49)_ = 6.37, *p* < 0.001. Detection sensitivity was lower for angry than for neutral faces, *t*_(49)_ = 3.40, *p* = 0.001.

#### Effects of task-relevant emotion in aging under cognitive load

Contrary to the hypothesis that the effect of task-relevant emotion will differ with age and load (hypothesis 2), the load × emotion × age group interaction in the expression task (see Table 3) was non-significant (*p* = 0.830).

#### Effects of task-irrelevant emotion in aging

There was a main effect of task-irrelevant emotion in the age task (see Table 3), as participants showed lower sensitivity when responding to the age of happy (*M* = 0.72, *SD* = 0.09) than of angry faces (*M* = 0.75, *SD* = 0.09), *t*_(49)_ = 2.53, *p* = 0.015. However, the difference in sensitivity between happy and neutral faces was non-significant (*p* = 0.057) and there was no difference between angry and neutral faces either (*p* = 0.576). Contrary to the hypothesis that the effects of task-irrelevant emotion will vary in the two age groups (hypothesis 3), the emotion × age group interaction in the age task was non-significant (*p* = 0.644).

### Reaction times

Load was successfully manipulated as evidenced by a main effect of load (see Table 2), with slower RTs in the 2-back (*M* = 1,056 ms, *SD* = 236) than in the 1-back task (*M* = 967 ms, *SD* = 150 ms). The five-way omnibus ANOVA revealed a task × emotion interaction, which qualified the main effect of emotion. The interaction was further qualified by a task × trial type × emotion interaction. These interactions were relevant for hypothesis 1, predicting a facilitating effect of task-relevant emotion. Separate analyses for RTs in the expression and age tasks were conducted to follow up these interactions (see Table 3).

#### Effects of task-relevant emotion

There was a trial type × emotion interaction in the expression task (see Table 3), but not in the age task. Separate analyses for match and non-match trials in the expression task revealed a main effect of emotion for match trials, *F*_(2, 96)_ = 57.18, *MSE* = 8,122, *p* < 0.001, partial η^2^ = 0.54, as RTs were faster for happy faces (*M* = 880 ms, *SD* = 198 ms) than for neutral (*M* = 970 ms, *SD* = 198 ms), *t*_(49)_ = 6.77, *p* < 0.001, or angry faces (*M* = 1,047 ms, *SD* = 199 ms), *t*_(49)_ = 11.99, *p* < 0.001. RTs were also faster for neutral than for angry faces, *t*_(49)_ = 5.51, *p* < 0.001. Analyses for non-match trials also revealed a main effect of emotion, *F*_(2, 96)_ = 7.36, *MSE* = 7,098, *p* = 0.001, partial η^2^ = 0.13. Similar to match trials, RTs on non-match trials were faster for happy faces (*M* = 1,020 ms, *SD* = 186 ms) than for neutral faces (*M* = 1,062 ms, *SD* = 206 ms), *t*_(49)_ = 3.26, *p* = 0.002. However, contrary to the pattern for match trials, RTs on non-match trials were also *faster* for angry faces (*M* = 1,005 ms, *SD* = 183 ms) than for neutral faces, *t*_(49)_ = 4.47, *p* < 0.001, with no difference between angry and happy faces (*p* = 0.117). Overall, these results are consistent with hypothesis 1, predicting a facilitating effect of task-relevant, positive emotion. Moreover, the results suggest that the effects of emotion on the actual updating process on non-match trials differ from those observed for match trials that do not require a replacement of old representations.

#### Effects of task-relevant emotion in aging under cognitive load

**Figure 3:**
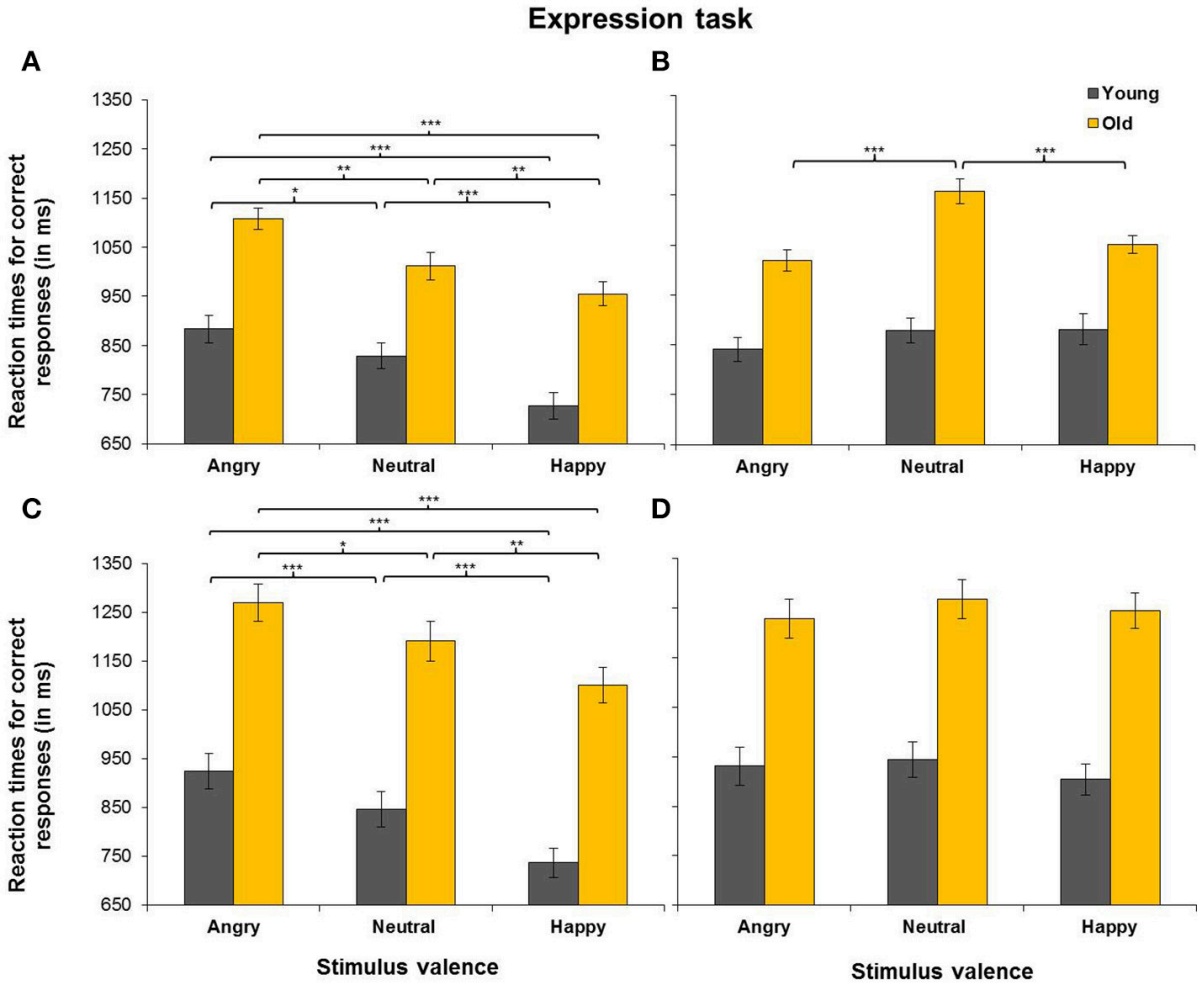
Reaction times for correct match responses **(A,C)** and non-match responses **(B,D)** in the expression task (i.e., participants responded to the emotional expression of faces). Presented are reaction times under 1-back load **(A,B)** and those under 2-back load **(C,D)**. Error bars represent SEM. ^***^p < 0.001, ^**^p < 0.01, ^*^p < 0.05.

**Figure 4:**
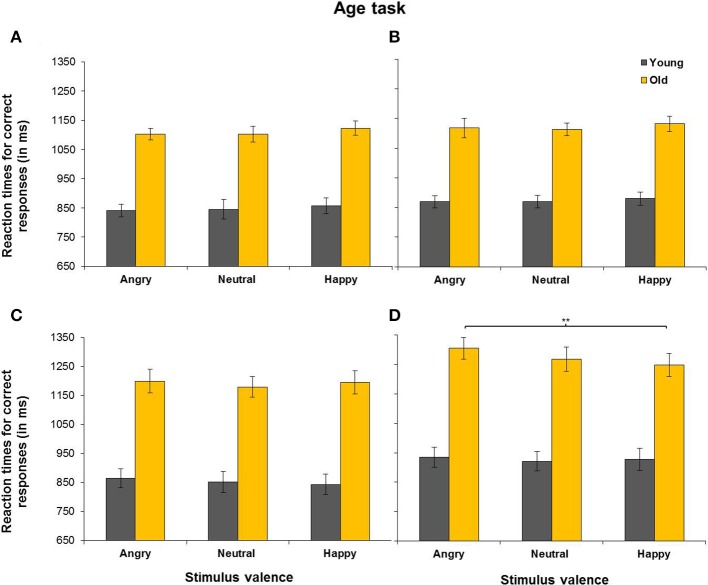
Reaction times for correct match responses **(A,C)** and non-match responses **(B,D)** in the age task (i.e., participants responded to the age of the faces). Presented are reaction times under 1-back load **(A,B)** and those under 2-back load **(C,D)**. Error bars represent SEM. ^**^p < 0.01.

The five-way ANOVA yielded no significant load × task × emotion × age group interaction, as per hypothesis 2. However, this interaction was part of a five-way load × task × trial type × emotion × age group interaction. Separate analyses for RTs in the expression and age tasks revealed that the load × trial type × emotion × age group interaction was significant in the expression task, but not in the age task. Separate analyses for 1-back and 2-back data in the expression task yielded a trial type × emotion × age interaction under 1-back conditions, *F*_(2, 96)_ = 7.78, *MSE* = 56,666, *p* = 0.001, partial η^2^ = 0.14, but not under 2-back conditions (*p* = 0.811). As a next step, only 1-back data were analyzed. Separate analyses for match and non-match trials in the expression task revealed an emotion × age group interaction for non-match trials, *F*_(2, 96)_ = 7.34, *MSE* = 6,102, *p* = 0.001, partial η^2^ = 0.13. In contrast, there was no such interaction for match trials (*p* = 0.336). For non-match trials, post-hoc *t*-tests revealed that older adults showed faster RTs for happy (*M* = 1,051 ms, *SD* = 92 ms) than for neutral faces (*M* = 1,157 ms, *SD* = 124 ms), *t*_(24)_ = 4.25, *p* < 0.001. RTs were also faster for angry (*M* = 1,019 ms, *SD* = 108 ms) than for neutral faces, *t*_(24)_ = 5.07, *p* < 0.001, with no difference in RTs for happy and angry faces (*p* = 0.066). In younger adults, the difference between RTs for angry and neutral faces was non-significant (*p* = 0.068) and there were no differences between RTs for happy and neutral faces (*p* = 0.930), or between RTs for angry and happy faces (*p* = 0.083). Thus, it appears that older adults' RTs for non-match trials were driving the trial type × emotion interaction reported above. RTs for match and non-match trials in the expression task across 1-back and 2-back loads are presented in Figure 3 and those in the age task are presented in Figure 4. In sum, the results suggest that older adults benefited from emotion to a higher extent than younger adults under low-load conditions as per hypothesis 2. However, this was only found for non-match trials, which suggest that emotion and age affected processes involved in the replacement of old representations.

#### Effects of task-irrelevant emotion in aging

As can be seen in Table 3, there was no effect of task-irrelevant emotion in the age task (*p* = 0.363) and contrary to the hypothesis that the effects of task-irrelevant emotion will differ in the two age groups (hypothesis 3), the emotion × age group interaction in the age task was non-significant (*p* = 0.806).

## Discussion

The present research investigated the facilitating and impairing effects of emotion on WM updating and showed that these vary with task relevance of emotion and with age. Task-relevant positive emotion generally facilitated performance in both age groups, with better detection sensitivity and faster RTs for happy compared to neutral or angry faces. However, whereas both age groups responded faster to task-relevant happy and slower to task-relevant angry faces on match trials, only older adults made faster non-match responses on trials with happy and angry relative to neutral faces. This suggests greater benefits from the inclusion of task-relevant emotion for older than younger adults when outdated material needs to be replaced in WM. Importantly, these benefits were observed when cognitive resources were available under low-load (1-back load) but not under high-load conditions (2-back load). In addition, it was found that task-irrelevant emotion did not impair WM updating as both age groups showed similar performance for emotional and neutral faces (despite lower detection sensitivity for happy relative to angry faces) when responding to the age of the faces.

### Facilitating effects of task-relevant emotion on WM updating

The finding that both age groups responded more accurately and faster to task-relevant happy relative to neutral or angry expressions supported the prediction that positive emotion would facilitate WM updating. It is also consistent with previous research showing faster updating of positive compared to neutral or negative stimuli (Levens and Gotlib, 2010, 2012; Pe et al., 2013a; Cromheeke and Mueller, 2015). Smiling faces improved performance across all trial types, which suggests that they facilitated general processes needed in the emotional *n*-task. One relevant mechanism could be the more efficient recognition of happy relative to other emotional expressions (Juth et al., 2005; Becker et al., 2011; Becker and Srinivasan, 2014). As reviewed by Becker and Srinivasan (2014), happy faces are efficiently recognized as they serve important social goals such as forestalling conflict. This advantage was observed across all loads in the present study, which supports research showing that the recognition of happy faces requires few cognitive resources (Srivastava and Srinivasan, 2010). An additional factor could be the facilitating effect of reward on executive functions. Happy faces were found to engage the orbitofrontal cortex, which is associated with reward (O'Doherty et al., 2003; Tsukiura and Cabeza, 2008). Reward, in turn, is believed to modulate cognitive control by fine-tuning executive functions needed for the task and by allocating additional resources (Pessoa, 2009, 2015, 2017). Thus, it is likely that the rewarding effect of a smile contributed to the happy face advantage in the *n*-back task. In contrast, participants showed lowest detection sensitivity for angry compared to neutral or happy faces and were also slowest when matching angry faces. These results are in line with findings that threatening material can impair WM performance (Pessoa, 2009, 2015, 2017). Despite this, beneficial effects of angry faces were also observed, which will be discussed below.

### Older adults benefit from task-relevant emotion more than younger adults

Crucially, this study showed that older adults benefited from task-relevant emotion to a greater extent than younger adults, which is in accordance with the study's predictions. Whereas both age groups made faster match responses to happy relative to neutral and angry faces, only older adults made faster non-match responses to emotional relative to neutral faces under low-load but not under high-load conditions. Importantly, emotion affected match and non-match responses differently and age-related differences emerged for non-match responses in the present study. These were generally slower than match responses, replicating previous findings (Verhaeghen and Basak, 2005; Chen et al., 2008).

It has been suggested that longer RTs on non-match trials can be attributed to the actual updating (i.e., replacement or overwriting) of outdated representations in WM, a process that is not needed on match trials (Verhaeghen and Basak, 2005). Thus, it appears that older adults benefited from task-relevant emotion when they had to perform this more complex non-match response. This finding indicates that they replaced old representations more readily with new emotional ones. However, a more detailed task analysis is needed to understand which sub-processes involved in non-match trials were particularly sensitive to the effects of aging and emotion. For instance, it is unclear whether the replacement of an old representation has been initiated or has already been achieved at the time of a button press. It is also unlikely that emotion recognition was driving the beneficial effect of emotion in non-match trials as this process is relevant for both match and non-match trials and as older adults usually show reduced recognition of angry faces (e.g., for a meta-analysis, see Ruffman et al., 2008; Krendl and Ambady, 2010). In contrast, it is possible that emotional faces facilitated responses on non-match trials in older adults as they were more distinct or salient compared to neutral faces. Given that older adults rely more on familiarity than on recollection relative to younger adults in WM updating (Schmiedek et al., 2009), emotion might improve their performance as they signal a non-match clearer than neutral items, whereas younger adults do not have to rely on these cues due to overall effective recollection processes. Further studies could help to elucidate which particular sub-processes of WM updating benefited particularly from task-relevant emotion in aging.

It is important to note that the facilitating effect of emotion on updating in older adults was only observed in the 1-back task when sufficient cognitive resources were available, but not in the 2-back task, where resources were depleted. This result might indicate that older adults used available resources in the 1-back task to focus on emotional items and that these did not facilitate performance in an “automatic” fashion. This interpretation is in accordance with SST, which suggests that older adults place greater importance on emotion compared to younger adults to enhance their well-being (Carstensen, 1993; Carstensen and Mikels, 2005; Reed and Carstensen, 2012). It is also consistent with evidence that older adults use cognitive resources to exhibit an emotion bias, which is eliminated by load (Mather and Knight, 2005).

The results obtained in the present study are consistent with previous research showing that WM performance in older adults can be improved when using emotional rather than neutral material (Mikels et al., 2005; Mammarella et al., 2013). However, as these prior studies focused on *maintenance* of content in WM only, the present study is the first to show that older adults can benefit from emotion in a WM task requiring the *updating* of information. Given research showing that aging is associated with impaired effectiveness of WM processes, particularly those requiring the manipulation of content in WM beyond its passive storage (Babcock and Salthouse, 1990; Salthouse, 1990, 1991; MacPherson et al., 2002; Zelazo et al., 2004; Reuter-Lorenz and Sylvester, 2005; Braver and West, 2008), the findings of the present study provide intriguing evidence that emotion can improve older adults' performance in more complex WM paradigms.

Possibly, the results might also help to shed light on high levels of well-being and efficient emotion regulation in aging (Gross et al., 1997; Carstensen et al., 2000; Blanchard-Fields, 2007; Larcom and Isaacowitz, 2009). Evidence suggests that the ability to update emotional information in WM is linked to the efficacy of emotion regulation (Levens and Gotlib, 2010; Pe et al., 2013a,b). Thus, it is possible that efficient updating of emotional content in WM is associated with high emotional control in aging. Future research should examine this link further to test whether older adults' ability to efficiently replace WM content with emotional information is associated with emotional control in aging.

### No age-related differences in effects of task-irrelevant emotion on updating

When participants were asked to respond to the age of the faces, updating was not affected to a greater extent by emotional relative to neutral expressions in either age group, suggesting that participants could focus on the on-going task in the presence of task-irrelevant emotion. This finding is in line with results of a previous study (Cromheeke and Mueller, 2015) showing that emotional expressions did not play a role when healthy adults had to updated the faces' gender. It is also compatible with research showing that emotional and neutral distractors do not affect updating differently (Mullin et al., 2012; Miendlarzewska et al., 2013; Ozawa et al., 2014).

Contrary to the hypotheses, older adults were not more susceptible to interference from task-irrelevant emotion than younger adults, which is in contrast to findings in previous WM studies (Wurm et al., 2004; Truong and Yang, 2014). Methodological differences might explain the divergent findings. For instance, Truong and Yang (2014) asked their participants to ignore distractors that were intermixed with targets. Each item was presented one at a time and participants were not able to predict whether the next word would be a target or distractor. Thus, they had to flexibly recruit different mechanisms such as inhibition of distractors and rehearsal of targets on a trial-by-trial basis. In the present research, however, participants were asked to respond to a non-emotional feature for the duration of an entire block, without having to engage in different strategies on different trials, which might have been less effortful for older adults.

Although there was no difference in detection sensitivity between emotional and neutral faces, sensitivity was lower for age decisions of happy relative to angry faces. It is possible that participants attended to the rewarding but task-irrelevant facial features to maximize reward, making them more susceptible to mistakes. Alternatively, sensitivity might have been lower due to difficulties in estimating the age of smiling faces. Previous studies found inaccurate age ratings for smiling faces (Voelkle et al., 2012; Ganel, 2015), which were attributed to factors such as wrinkles around the eyes (Ganel, 2015) or to stereotypes linking happiness with youth (Voelkle et al., 2012). It is possible that these factors contributed to “noise” when participants responded to happy compared to angry faces, which reduced sensitivity during the updating of the faces' age.

## Conclusion

In sum, the study contributed to research differentiating between enhancing and impairing effects of emotion on WM updating in younger and older adults. When emotion was task-relevant, happy faces improved updating performance in both age groups, which is in line with previous research. Crucially, this study extended previous research by showing that older adults benefited to a greater extent than younger adults from the inclusion of emotional material in a complex WM task and that they were not more susceptible to interference from task-irrelevant emotion. This research is important as understanding the facilitating effects of emotion on cognition in aging can help identifying areas in which preserved emotional processing can help buffering age-related WM decline. Moreover, further investigation of older adults' successful manipulation of emotional material in WM can contribute to our understanding of high well-being in aging, as being able to efficiently update emotional material in WM might facilitate emotion control.

## Author contributions

NB: Conception and design of the work, data collection, data analysis, and interpretation, drafting of the manuscript. AR and ED: Contribution to conception and design of the work, the interpretation of the data, critical revision of the manuscript.

### Conflict of interest statement

The authors declare that the research was conducted in the absence of any commercial or financial relationships that could be construed as a potential conflict of interest. The reviewer EP and handling Editor declared their shared affiliation, and the handling Editor states that the process nevertheless met the standards of a fair and objective review.
