# Supplementary material for: When Emotions Matter: Focusing on Emotion Improves Working Memory Updating in Older Adults
Source: Front Psychol. 2017 Sep 15;8:1565. doi: 10.3389/fpsyg.2017.01565 (PMC5605649; doi:10.3389/fpsyg.2017.01565)
Supplement: Supplementary file 1 [file Table1.doc]

## Supplemental material

***Evaluation of the stimulus set used in the main experiment***

Ten younger (21 – 32 years old; *M* = 27.80, SD = 3.12) and ten older adults (66 – 76 years old; *M* = 71.27, *SD* = 3.13) rated 234 preselected faces from the FACES database (Ebner, Riediger, & Lindenberger, 2010). Seventy-eight faces per emotional category (happy, neutral, angry) were included and one-third of the faces were from young adults (19–31 years), one-third from middle-aged adults (39–55 years) and the remaining faces from older adults (69–80 years). Half of the faces were male, the other half were female. The faces were rated on a 9-point Likert scale regarding valence (1 = *very unpleasant*; 9 = *very pleasant*) and arousal (1 = *not arousing*; 9 = *very arousing*) and raters were also asked to estimate the age of the presented face. Valence and arousal ratings were ranked according to difference scores of younger and older adults’ ratings. Data from this rating study indicated that all happy faces (with the exception of one face) were rated as being more pleasant by older relative to younger raters, which was also reflected in marginally significant higher valence ratings for those faces in older compared to younger adults (*p* = .051).

From these 234 faces, 72 faces with the highest agreement between younger and older raters were selected for the main experiment (see Table 1 and Table 2 for ratings). Twenty-four showed a happy expression, 24 showed a neutral expression and the remaining 24 showed an angry expression. Faces were selected such that both age group and sex of the face models were balanced in each emotion category, resulting in eight pictures per age group and emotion category with four pictures showing male faces and four pictures showing female faces. Each picture showed a unique individual.

To create two face sets for counterbalancing purposes, 12 neutral, 12 happy, and 12 angry faces were allocated to Set A and the remaining 12 neutral, 12 happy, and 12 angry faces were allocated to Set B. The faces in Set A and Set B did not differ significantly in terms of arousal, *t*(19) = .07, *p* = .948, ns, or valence, *t*(19) = 1.30, *p* = .208, ns.

## Tables

| Table 1. *Age ratings.* | | | | | | | | | |
| --- | --- | --- | --- | --- | --- | --- | --- | --- | --- |
|  |  | Age group of face | | | | | | | |
|  | Emotional expression of face | Young | |  | Middle-aged | |  | Old | |
|  |  | *M* | *SD* |  | *M* | *SD* |  | *M* | *SD* |
|  |  |  | |  |  | |  |  | |
| Younger raters | Happy | 23.3 | 2.4 |  | 45.0 | 3.25 |  | 68.9 | 5.8 |
|  | Neutral | 25.2 | 1.6 |  | 44.8 | 5.3 |  | 69.8 | 5.7 |
|  | Angry | 25.6 | 2.3 |  | 45.6 | 3.5 |  | 67.6 | 6.1 |
|  |  |  |  |  |  |  |  |  |  |
| Older raters | Happy | 27.1 | 4.2 |  | 46.6 | 2.4 |  | 69.8 | 4.8 |
|  | Neutral | 26.3 | 2.5 |  | 45.2 | 3.9 |  | 73.3 | 4.6 |
|  | Angry | 27.2 | 3.2 |  | 44.9 | 3.3 |  | 70.1 | 4.2 |
|  |  |  |  |  |  |  |  |  |  |

| Table 2. *Arousal and valence ratings.* | | | | | | | | |
| --- | --- | --- | --- | --- | --- | --- | --- | --- |
|  | Emotional expression of face | | | | | | | |
|  | Happy | |  | Neutral | |  | Angry | |
|  | *M* | *SD* |  | *M* | *SD* |  | *M* | *SD* |
|  |  | |  |  | |  |  | |
| Arousal ratings |  |  |  |  |  |  |  |  |
| Younger raters | 6.29 | .64 |  | 4.39 | 1.19 |  | 6.76 | .81 |
| Older raters | 6.77 | 1.75 |  | 4.91 | 1.10 |  | 7.22 | 1.28 |
|  |  |  |  |  |  |  |  |  |
| Valence ratings |  |  |  |  |  |  |  |  |
| Younger raters | 6.84 | 1.02 |  | 4.70 | .40 |  | 2.52 | .60 |
| Older raters | 8.03 | .63 |  | 4.83 | .50 |  | 2.38 | .60 |
|  |  |  |  |  |  |  |  |  |
